# Supplementary material for: Functional and antigenic characterization of SARS-CoV-2 spike fusion peptide by deep mutational scanning
Source: Nat Commun. 2024 May 14;15:4056. doi: 10.1038/s41467-024-48104-8 (PMC11094058; doi:10.1038/s41467-024-48104-8)
Supplement: Supplementary file 1 — Supplementary Information [file 41467_2024_48104_MOESM1_ESM.pdf]

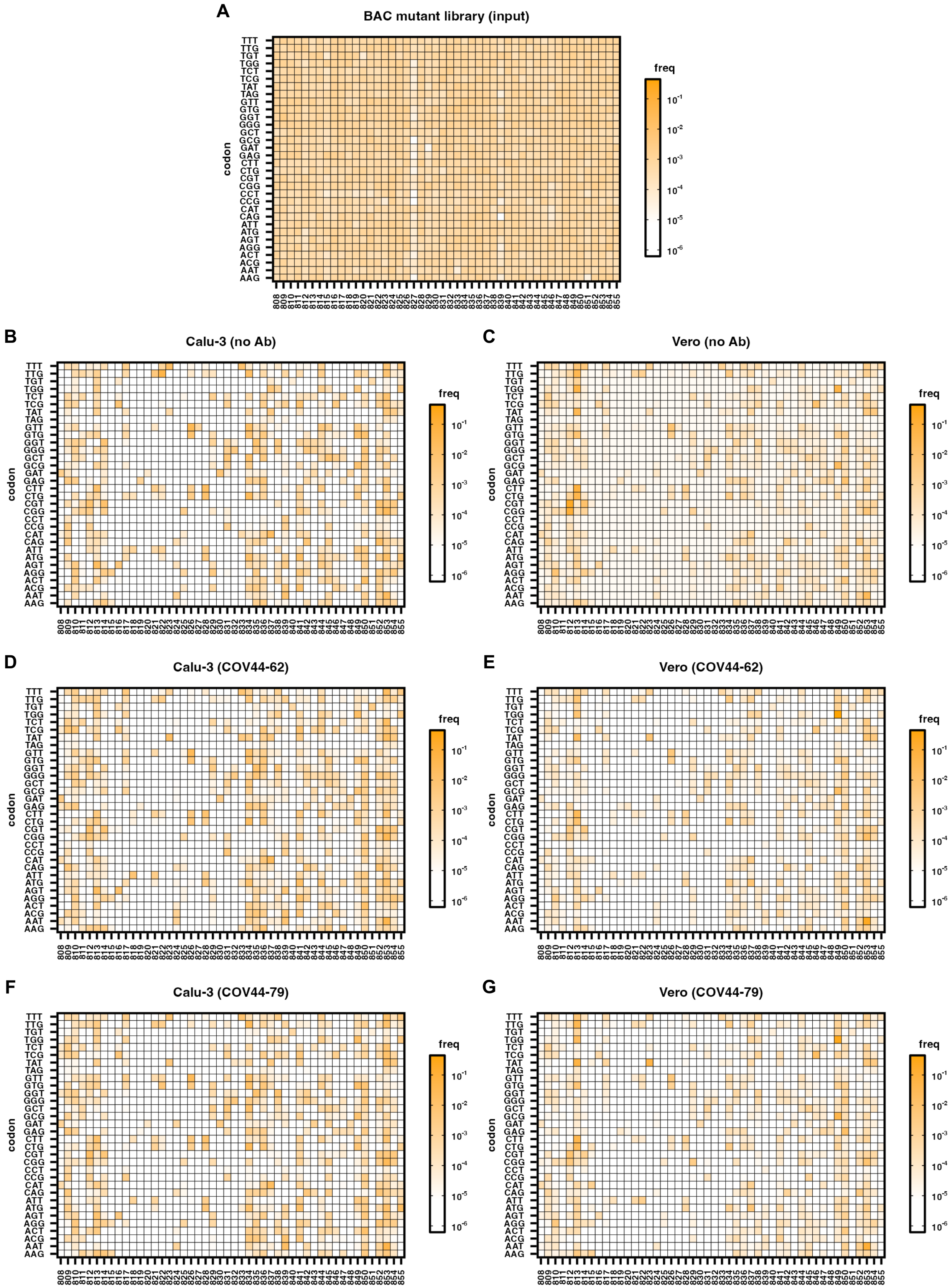

A
BAC mutant library (input)
B
Calu-3 (no Ab)
C
Vero (no Ab)
D
Calu-3 (COV44-62)
E
Vero (COV44-62)
F
Calu-3 (COV44-79)
G
Vero (COV44-79)
codon
freq
TTT TTG TGT TGG TCT TCG TAT TAG GTT GTG GGT GGG GCT GCG GAT GAG CTT CTG CGT CGG CCT CCG CAT CAG ATT ATG AGT AGG ACT ACG AAT AAG
808 809 810 811 812 813 814 815 816 817 818 819 820 821 822 823 824 825 826 827 828 829 830 831 832 833 834 835 836 837 838 839 840 841 842 843 844 845 846 847 848 849 850 851 852 853 854 855

Figure S1. Heatmaps showing the frequencies of individual codon variants.

(**A**) Frequency of different codon variants in the SARS-CoV-2 BAC DNA libraries. (**B-G**) Frequency of different codon variants in the virus mutant library after one passage in Calu-3 cells (**B**), Vero cells (**C**), Calu-3 cells with COV44-62 (**D**), Vero cells with COV44-62 (**E**), Calu-3 cells with COV44-79 (**F**) and Vero cells with COV44-79. All frequency values shown in the heatmaps are averages of the two independent replicates.

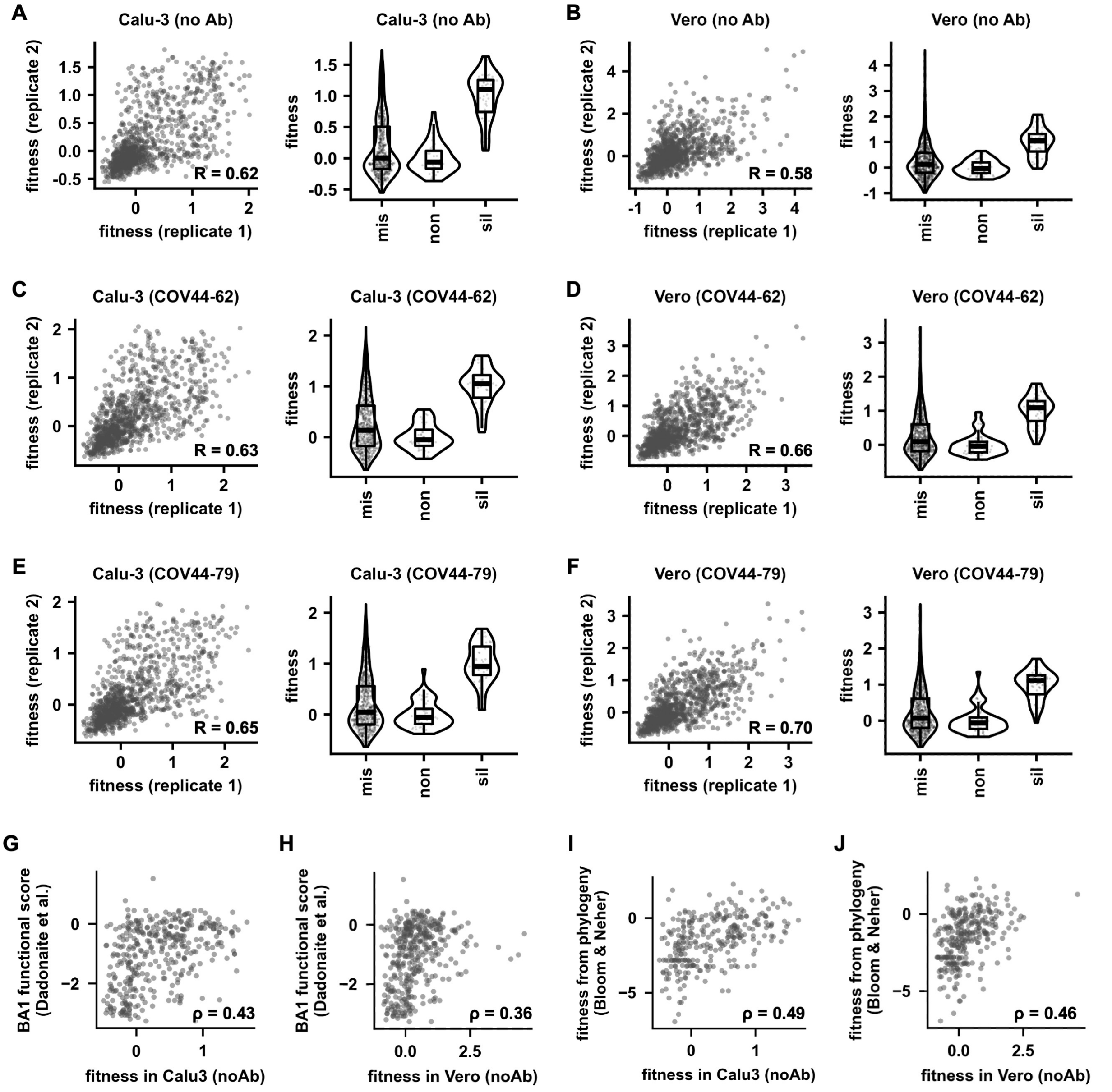

Figure S2. Reproducibility and quality of the deep mutational scanning data. Correlation of fitness values for individual mutations between two biological replicates is shown as a scatterplot. Pearson correlation coefficients (R) are indicated (left panel). The distributions of fitness values for missense mutations (mis), nonsense mutations (non), and silent mutations (sil) are shown as a stripchart overlayed with a violin plot (right panel). Of note, silent mutations represent nucleotide variants that encode the WT amino-acid sequences but were different from the WT nucleotide sequence. Deep mutational scanning was performed in six conditions, namely **(A)** Calu-3 cells

with no antibody selection, **(B)** Vero cells with no antibody selection, **(C)** Calu-3 cells with COV44-62 antibody selection, **(D)** Vero cells with COV44-62 antibody selection, **(E)** Calu-3 cells with COV44-79 antibody selection, and **(F)** Vero cells with COV44-79 antibody selection. **(G-H)** Previously, Dadonaite et al. measured the effects of mutations in BA.1 S on virus entry [S1]. Correlations between the functional scores reported by Dadonaite et al. [S1] and **(G)** fitness values in Calu-3 cells, or **(H)** Vero cells are shown. **(I-J)** Previously, Bloom and Neher computed the fitness effects of mutations to all SARS-CoV-2 proteins using a phylogeny-based approach [S2]. Correlations between the fitness computed by Bloom and Neher [S2] and **(I)** fitness values in Calu-3 cells, or **(J)** Vero cells are shown. **(G-I)** Only missense mutations were analyzed. Spearman's rank correlation coefficients (ρ) are indicated.

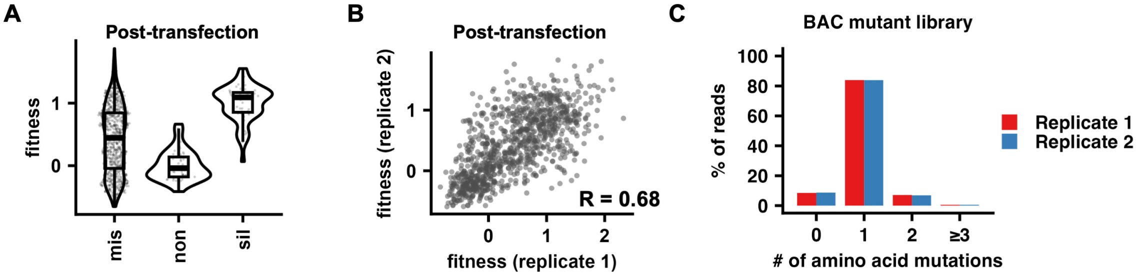

D

```
                                                                # of AA mut
WT        DPSKPSKRSFIEDLLFNKVTLADAGFIKQYGDCLGDIAARDLICAQKF         0
Clone1    DPSKPSKRSFIEDLLFNKVTLADAGFIKQYGDCLGDIAARDLICAQKF         0
Clone2    DPSKPSKRSFIEDLLFNKVTLADAGFIKQYGDVLGDIAARDLICAQKF         1
Clone3    DPSKPSKRSFIEDLLFNKVTLADAGFIKQYGDCLGDIAARDLICAQKF         0
Clone4    DPSKPSKRSFIEDLLFNKVTLADAGFIKQYGDCLGDIAARDLDCAQKF         1
Clone5    DPSKPSKRSFIEDLLFNKVTLADAGFIKGYGDCLGDIAARDLICAQKF         1
Clone6    DPSKPSKRSFIEDLLFNLVTLADAGFIKQYGDCLGDIAARDLICAQKF         1
Clone7    DPSKPSKRSFIEDLLFNKVTLADAGFIKQYGDCLGDIAARDLICAQKF         1
Clone8    DPS*PSKRSFIEDLLFNKVTLADAGFIKQYGDCLGDIAARDLICAQKF         1
Clone9    DPSKPSKRSFIEDLLFNKVTLWDAGFIKQYGDCLGDIAARDLICARKF         2
Clone10   DPSKPSKRSFIEDLLFNKVTLADAGFIKQYGDCLLDIAARDLICAQKF         1
Clone11   DPSKPSKRSFIEDLLFNKVTLADAGFIKQYGDCLGDIAARDLICAQKF         0
Clone12   DPSKPSKRSFIEDLLFNKVTLADAGFIKQYGDCLGDIAARDLICAQKF         0
Clone13   DPSKPSKRSSIEDLLFNKVTLADAGFIKQYGDCLGDIAARDLICAQKF         1
Clone14   DPSKPSKRSFIEDLLFNKVTLADAGFIKQYGDCLGDWAARDLICAQKF         1
Clone15   DPSKPSKRSFIEDLLFNKVTLADAGFRKQYGDCLGDIAARDLICAQKF         1
Clone16   DPSKPSKRSFIETLLFNKVTLADAGFIKQYGDCLGDIAARDLICAQKF         1
Clone17   DPSKPSKRSFMEDLLFNKVTLADAGFIKQGGDCLGDIAARDLICAQKF         2
Clone18   DPSKPSKRSFIEDLLFNKVTLADAGFIKQYGDCLGDIAARDLICAQKF         1
Clone19   DPSKPSKRSFIEDLLFNKNTLADAGFIKQYGDCLGDIAARDLICAQKF         1
Clone20   DPSKPSKRSFIEDLLFNKVTLADAGFIKQYGDCQGDIAARDLICAQKF         1
Clone21   DPSKPSKRSFIEDLLFNKVTLADAGFIKQYGDCLGDIWARDLICAQKF         1
Clone22   DPSKPLKRSFIEDLLFNKVTLADAGFIKQYGDCLGDIAARDLICAQKF         1
```

Figure S3. Analysis of the number of mutations per clone in the input DNA library. (A) The distributions of post-transfection fitness values for missense mutations (mis), nonsense mutations (non), and silent mutations (sil) are shown as a stripchart overlayed with a violin plot (left panel). Of note, silent mutations represent nucleotide variants that encode the WT amino-acid sequences but were different from the WT nucleotide sequence. Two-tailed t-tests indicated that the distributions of post-transfection fitness values for these three types of mutations significantly differ ($p < 0.0001$). **(B)** Correlation of post-transfection fitness values for individual mutations between two biological replicates is shown as a scatterplot (right panel). Pearson correlation

coefficients (R) are indicated. **(C)** Paired-end reads were merged (**see Methods**), translated, and compared to the WT amino acid sequence. The number of amino acid mutations of each paired-end reads was counted. Percentage of reads (y-axis) with the indicated number of amino acid mutations (x-axis) are shown. **(D)** Sequence alignment of 22 individual clones randomly selected from the BAC mutant library for Sanger sequencing. “WT” sequence refers to residue 808 to 855 of the spike protein in Wuhan-Hu-1 strain. “# of AA mut” refers to the number of amino acid mutations in each clone compared to the WT sequence.

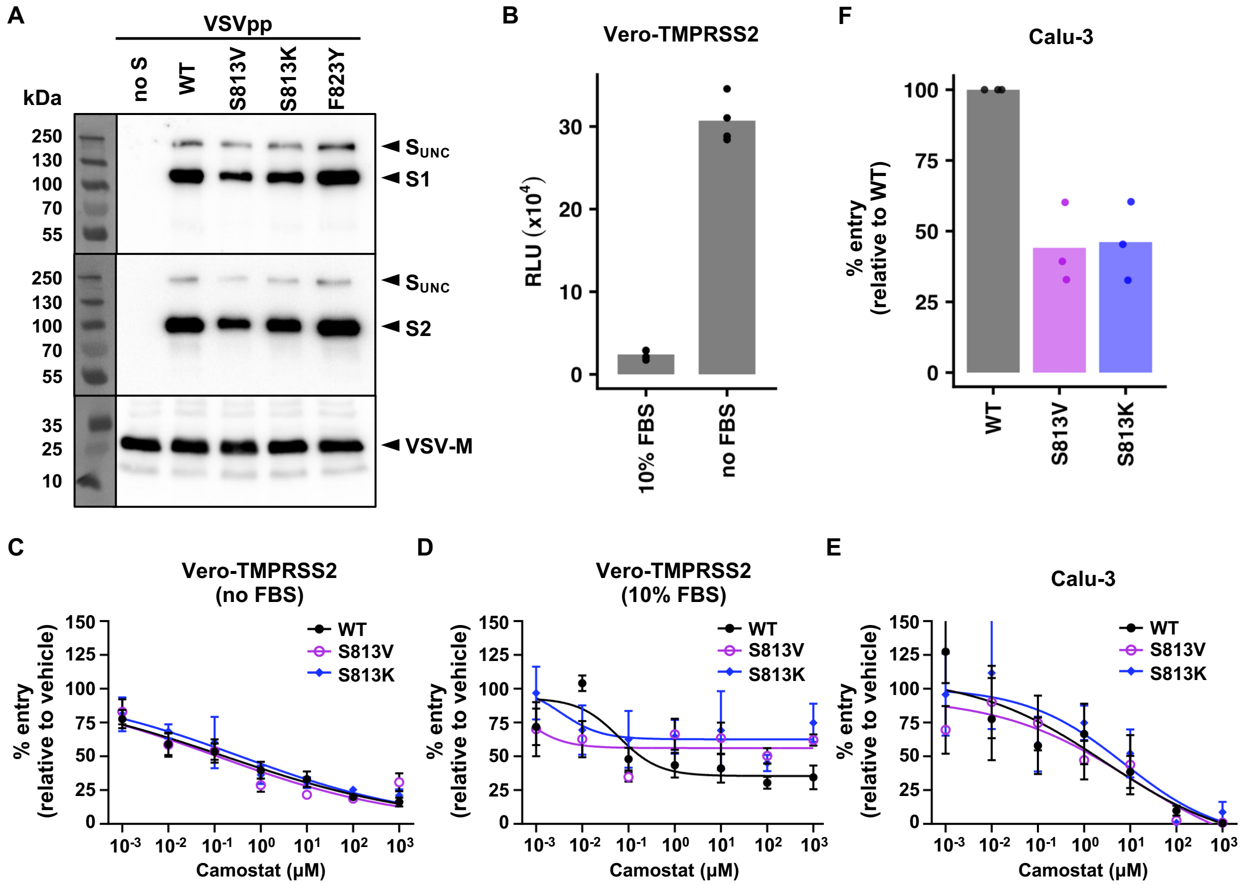

Figure S4. Characterizing SARS-CoV-2 S mutations using VSVpps. (A) Western blot analysis of VSVpps bearing various S constructs. Uncleaved S (S_{UNC}), S1, S2, and VSV-M are labeled. **(B)** Vero-TMPRSS2 cell entry in the absence or presence of FBS by VSVpps bearing SARS-CoV-2 S (WT) was measured by the relative light unit (RLU) in a luciferase assay. Each bar represents the mean of four biological replicates. Each datapoint represents one biological replicate. **(C and D)** The effects of camostat on Vero-TMPRSS2 cell entry of VSVpps bearing various SARS-CoV-2 S constructs, **(C)** in the absence or **(D)** presence of FBS. **(E)** The effects of camostat on Calu-3 cell entry of VSVpps bearing various SARS-CoV-2 S constructs. (**F**). Entry in Calu-3 cells of VSVpps bearing various SARS-CoV-2 S constructs normalized to SARS-CoV-2 S (WT). Mean and SEM of four biological replicates are depicted.

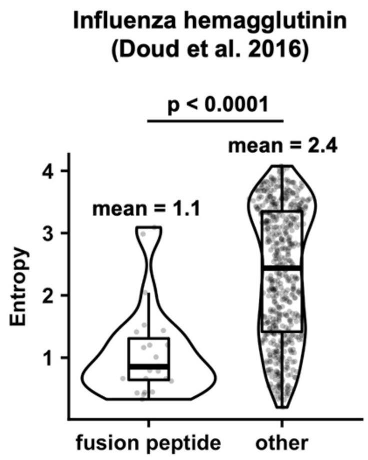

Figure S5. Mutational tolerance of influenza hemagglutinin fusion peptide. A previous deep mutational scanning study reported the mutational tolerance of each amino acid residue in the influenza H1N1 hemagglutinin [S3]. This previous study quantified mutational tolerance of each amino acid residue as entropy. A lower entropy value indicates lower mutational tolerance. The distributions of entropy for the 23 residues in the fusion peptide of influenza hemagglutinin and non-fusion peptide residues are shown as a stripchart overlayed with a violin plot. The indicated p-value is computed by Wilcoxon rank-sum test.

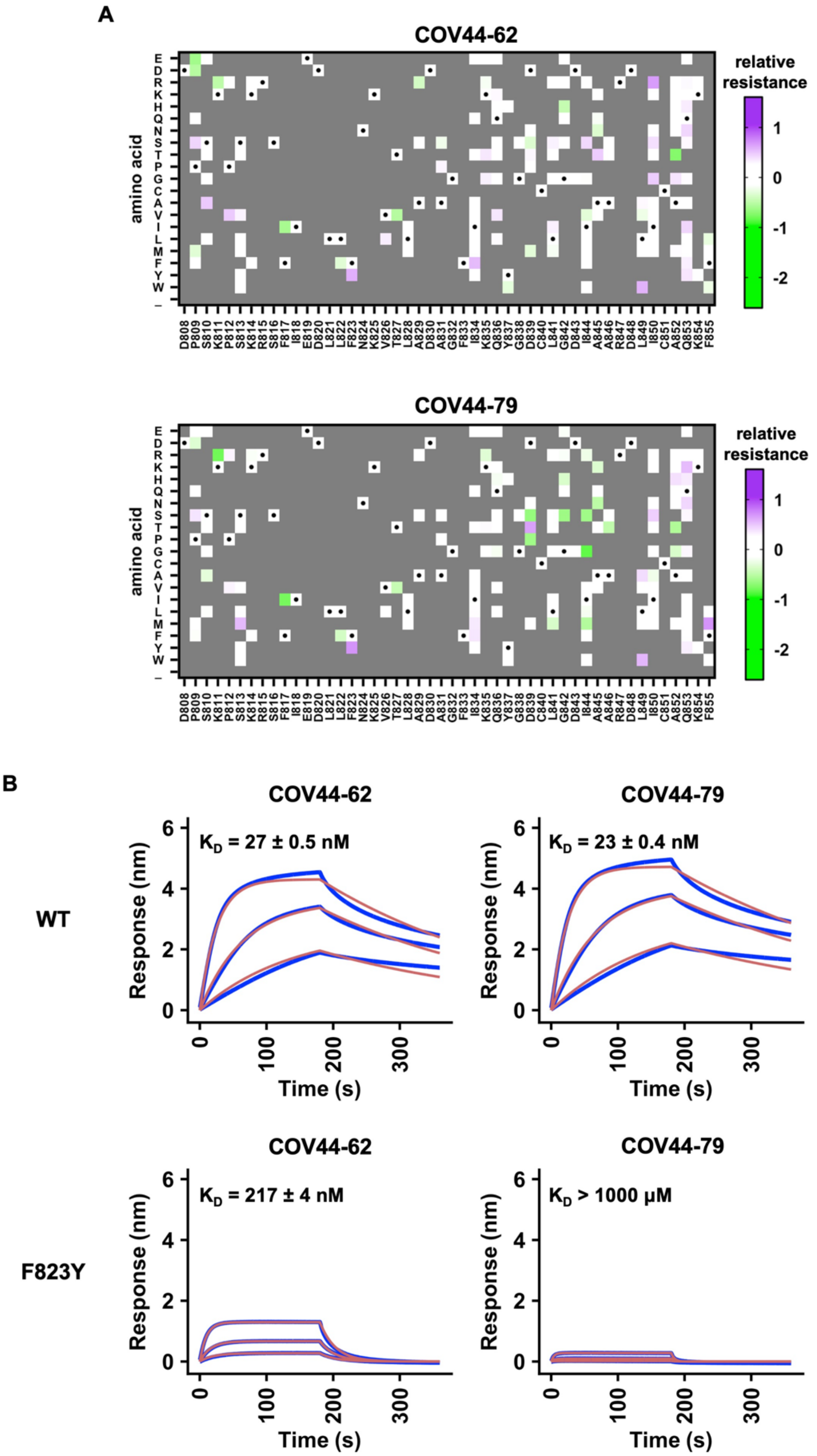

Figure S6. F823Y mutation weakens the binding of bFP antibodies. (A) Relative resistance for each mutation against 230 μg/mL COV44-62 antibody or 330 μg/mL COV44-79 antibody in

Calu-3 cells is shown as heatmaps. Relative resistance for WT is set as 0. Mutations with a fitness value of less than 0.75 are shown as gray. Amino acids corresponding to the WT sequence are indicated by the black dots. **(B)** Binding kinetics of COV44-62 Fab or COV44-79 Fab against WT or F823Y peptide that contained residues 808 to 827 were measured by biolayer interferometry (BLI). Y-axis represents the response. Blue lines represent the response curve and red lines represent the 1:1 binding model. Binding kinetics were measured for three concentrations of Fab at 3-fold dilution ranging from 300 nM to 33.3 nM.

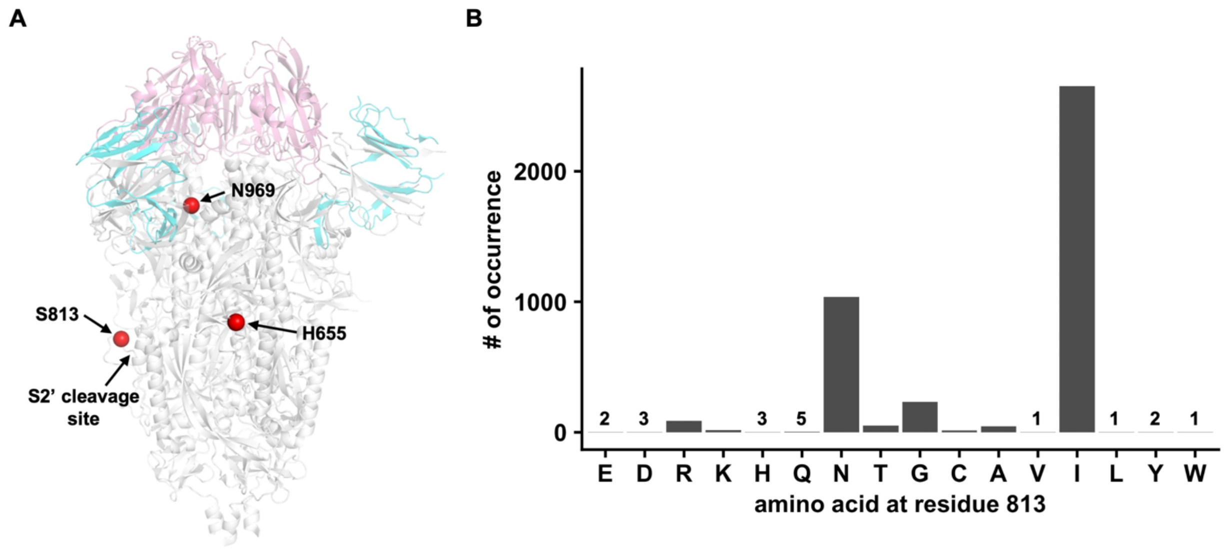

Figure S7. Frequency of natural mutations at residue 813. (A) The Cαs of residues 655, 813 and 969 are shown in red spheres on the SARS-CoV-2 spike structure (PDB 6VXX) [S4]. **(B)** Occurrences of different amino acid mutations at residue 813 among 15 million SARS-CoV-2 genomes on GISAID are shown. The wild-type variant Ser (S) is not shown. Occurrence of less than 10 is indicated.

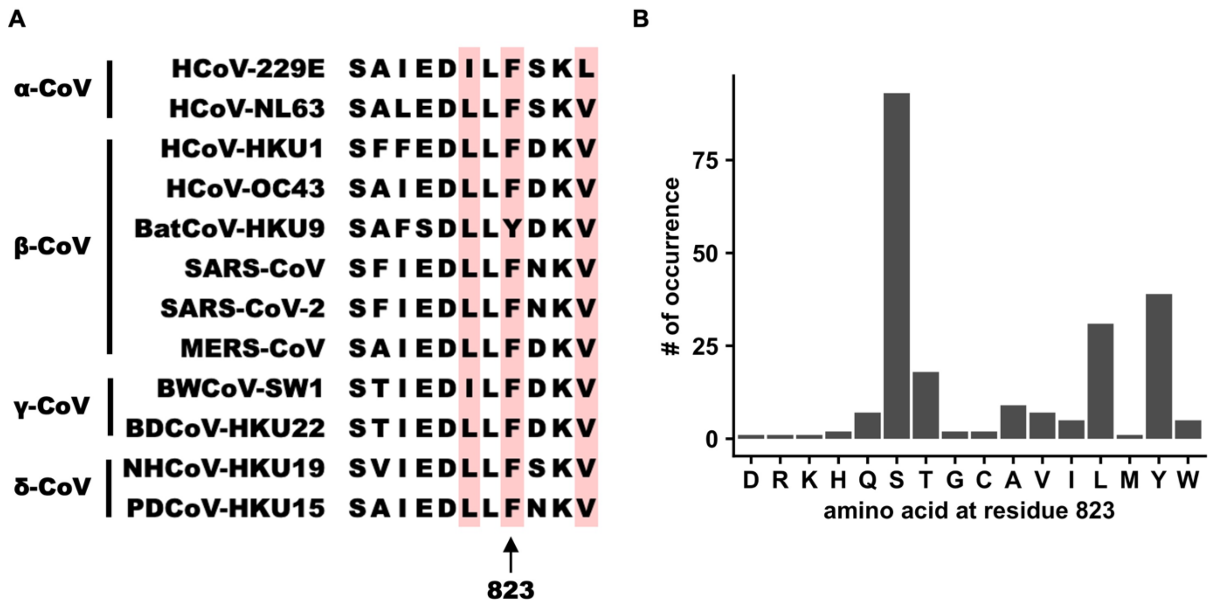

Figure S8. Natural occurrence of F823Y. (A) Multiple sequence alignment of the first 11 residues of bFP from different strains that represent four coronavirus subgroups (α, β, γ, and δ). Residues that are not completely conserved among these sequences are highlighted in pink. **(B)** Occurrences of different amino acid mutations at residue 823 among 15 million SARS-CoV-2 genomes on GISAID are shown. The wild-type variant Phe (F) is not shown.

Name	Type	Sequence
Cassette1_1	Forward	5'-AAT TTT TCA CAA ATA TTA CCA NNK CCT TCT AAA CCA AGC AAG AGG TCA TTT ATT GAA GAT-3'
Cassette1_2	Forward	5'-AAT TTT TCA CAA ATA TTA CCA GAC NNK TCA AAG CCA AGC AAG AGG TCA TTT ATT GAA GAT-3'
Cassette1_3	Forward	5'-AAT TTT TCA CAA ATA TTA CCA GAT CCT NNK AAG CCT AGC AAG AGG TCA TTT ATT GAA GAT-3'
Cassette1_4	Forward	5'-AAT TTT TCA CAA ATA TTA CCA GAC CCA TCT NNK CCT AGC AAG AGG TCA TTT ATT GAA GAT-3'
Cassette1_5	Forward	5'-AAT TTT TCA CAA ATA TTA CCA GAT CCT TCA AAA NNK AGT AAG AGG TCA TTT ATT GAA GAT-3'
Cassette1_6	Forward	5'-AAT TTT TCA CAA ATA TTA CCA GAT CCA TCT AAG CCA NNK AAG AGG TCA TTT ATT GAA GAT-3'
Cassette1_7	Forward	5'-AAT TTT TCA CAA ATA TTA CCA GAC CCT TCA AAA CCT AGC NNK AGG TCA TTT ATT GAA GAT-3'
Cassette1_8	Forward	5'-AAT TTT TCA CAA ATA TTA CCA GAT CCA TCT AAA CCT AGT AAG NNK TCA TTT ATT GAA GAT-3'
Cassette2_1	Forward	5'-CCA TCA AAA CCA AGC AAG AGG NNK TTC ATC GAA GAT CTA CTT TTC AAC AAA GTG ACA CTT-3'
Cassette2_2	Forward	5'-CCA TCA AAA CCA AGC AAG AGG TCT NNK ATT GAG GAT CTA CTT TTC AAC AAA GTG ACA CTT-3'
Cassette2_3	Forward	5'-CCA TCA AAA CCA AGC AAG AGG TCT TTT NNK GAA GAC CTA CTT TTC AAC AAA GTG ACA CTT-3'
Cassette2_4	Forward	5'-CCA TCA AAA CCA AGC AAG AGG TCA TTC ATT NNK GAC CTA CTT TTC AAC AAA GTG ACA CTT-3'
Cassette2_5	Forward	5'-CCA TCA AAA CCA AGC AAG AGG TCA TTT ATC GAG NNK CTA CTT TTC AAC AAA GTG ACA CTT-3'
Cassette2_6	Forward	5'-CCA TCA AAA CCA AGC AAG AGG TCT TTC ATC GAG GAC NNK CTT TTC AAC AAA GTG ACA CTT-3'
Cassette2_7	Forward	5'-CCA TCA AAA CCA AGC AAG AGG TCT TTT ATC GAG GAT CTC NNK TTC AAC AAA GTG ACA CTT-3'
Cassette2_8	Forward	5'-CCA TCA AAA CCA AGC AAG AGG TCT TTT ATT GAG GAC CTC CTT NNK AAC AAA GTG ACA CTT-3'
Cassette3_1	Forward	5'-TTT ATT GAA GAT CTA CTT TTC NNK AAG GTA ACA CTT GCA GAT GCT GGC TTC ATC AAA CAA-3'
Cassette3_2	Forward	5'-TTT ATT GAA GAT CTA CTT TTC AAT NNK GTG ACC CTT GCA GAT GCT GGC TTC ATC AAA CAA-3'
Cassette3_3	Forward	5'-TTT ATT GAA GAT CTA CTT TTC AAC AAG NNK ACC CTC GCA GAT GCT GGC TTC ATC AAA CAA-3'
Cassette3_4	Forward	5'-TTT ATT GAA GAT CTA CTT TTC AAT AAA GTA NNK CTC GCA GAT GCT GGC TTC ATC AAA CAA-3'
Cassette3_5	Forward	5'-TTT ATT GAA GAT CTA CTT TTC AAC AAG GTG ACA NNK GCC GAT GCT GGC TTC ATC AAA CAA-3'
Cassette3_6	Forward	5'-TTT ATT GAA GAT CTA CTT TTC AAC AAA GTA ACC CTT NNK GAT GCT GGC TTC ATC AAA CAA-3'
Cassette3_7	Forward	5'-TTT ATT GAA GAT CTA CTT TTC AAT AAG GTG ACA CTC GCA NNK GCT GGC TTC ATC AAA CAA-3'
Cassette3_8	Forward	5'-TTT ATT GAA GAT CTA CTT TTC AAC AAA GTA ACA CTC GCC GAT NNK GGC TTC ATC AAA CAA-3'
Cassette4_1	Forward	5'-AAA GTG ACA CTT GCA GAT GCT NNK TTT ATA AAA CAA TAT GGT GAT TGC CTT GGT GAT ATT-3'
Cassette4_2	Forward	5'-AAA GTG ACA CTT GCA GAT GCT GGT NNK ATC AAG CAA TAT GGT GAT TGC CTT GGT GAT ATT-3'

Cassette4_3	Forward	5'-AAA GTG ACA CTT GCA GAT GCT GGT TTC NNK AAA CAG TAT GGT GAT TGC CTT GGT GAT ATT-3'
Cassette4_4	Forward	5'-AAA GTG ACA CTT GCA GAT GCT GGC TTT ATC NNK CAG TAT GGT GAT TGC CTT GGT GAT ATT-3'
Cassette4_5	Forward	5'-AAA GTG ACA CTT GCA GAT GCT GGC TTC ATA AAG NNK TAT GGT GAT TGC CTT GGT GAT ATT-3'
Cassette4_6	Forward	5'-AAA GTG ACA CTT GCA GAT GCT GGT TTT ATA AAG CAG NNK GGT GAT TGC CTT GGT GAT ATT-3'
Cassette4_7	Forward	5'-AAA GTG ACA CTT GCA GAT GCT GGT TTT ATC AAA CAA TAC NNK GAT TGC CTT GGT GAT ATT-3'
Cassette4_8	Forward	5'-AAA GTG ACA CTT GCA GAT GCT GGT TTC ATC AAG CAG TAC GGT NNK TGC CTT GGT GAT ATT-3'
Cassette5_1	Forward	5'-TTC ATC AAA CAA TAT GGT GAT NNK CTC GGG GAT ATT GCT GCT AGA GAC CTC ATT TGT GCA-3'
Cassette5_2	Forward	5'-TTC ATC AAA CAA TAT GGT GAT TGT NNK GGT GAC ATT GCT GCT AGA GAC CTC ATT TGT GCA-3'
Cassette5_3	Forward	5'-TTC ATC AAA CAA TAT GGT GAT TGT CTT NNK GAT ATC GCT GCT AGA GAC CTC ATT TGT GCA-3'
Cassette5_4	Forward	5'-TTC ATC AAA CAA TAT GGT GAT TGC CTC GGT NNK ATC GCT GCT AGA GAC CTC ATT TGT GCA-3'
Cassette5_5	Forward	5'-TTC ATC AAA CAA TAT GGT GAT TGC CTT GGG GAC NNK GCT GCT AGA GAC CTC ATT TGT GCA-3'
Cassette5_6	Forward	5'-TTC ATC AAA CAA TAT GGT GAT TGT CTC GGG GAC ATC NNK GCT AGA GAC CTC ATT TGT GCA-3'
Cassette5_7	Forward	5'-TTC ATC AAA CAA TAT GGT GAT TGT CTC GGT GAT ATC GCA NNK AGA GAC CTC ATT TGT GCA-3'
Cassette5_8	Forward	5'-TTC ATC AAA CAA TAT GGT GAT TGT CTT GGT GAT ATT GCA GCT NNK GAC CTC ATT TGT GCA-3'
Cassette6_1	Forward	5'-CTT GGT GAT ATT GCT GCT AGA NNK CTT ATC TGT GCA CAA AAG TTT AAC GGC CTT ACT GTT-3'
Cassette6_2	Forward	5'-CTT GGT GAT ATT GCT GCT AGA GAT NNK ATT TGC GCA CAA AAG TTT AAC GGC CTT ACT GTT-3'
Cassette6_3	Forward	5'-CTT GGT GAT ATT GCT GCT AGA GAT CTC NNK TGT GCC CAA AAG TTT AAC GGC CTT ACT GTT-3'
Cassette6_4	Forward	5'-CTT GGT GAT ATT GCT GCT AGA GAC CTT ATT NNK GCC CAA AAG TTT AAC GGC CTT ACT GTT-3'
Cassette6_5	Forward	5'-CTT GGT GAT ATT GCT GCT AGA GAC CTC ATC TGC NNK CAA AAG TTT AAC GGC CTT ACT GTT-3'
Cassette6_6	Forward	5'-CTT GGT GAT ATT GCT GCT AGA GAT CTT ATC TGC GCC NNK AAG TTT AAC GGC CTT ACT GTT-3'
Cassette6_7	Forward	5'-CTT GGT GAT ATT GCT GCT AGA GAT CTC ATC TGT GCA CAG NNK TTT AAC GGC CTT ACT GTT-3'
Cassette6_8	Forward	5'-CTT GGT GAT ATT GCT GCT AGA GAT CTC ATT TGC GCC CAG AAG NNK AAC GGC CTT ACT GTT-3'
Cassette1_Rprimer	Reverse	5'-TGG TAA TAT TTG TGA AAA ATT-3'
Cassette2_Rprimer	Reverse	5'-CCT CTT GCT TGG TTT TGA TGG-3'
Cassette3_Rprimer	Reverse	5'-GAA AAG TAG ATC TTC AAT AAA-3'

Cassette4_Rprimer	Reverse	5'-AGC ATC TGC AAG TGT CAC TTT-3'
Cassette5_Rprimer	Reverse	5'-ATC ACC ATA TTG TTT GAT GAA-3'
Cassette6_Rprimer	Reverse	5'-TCT AGC AGC AAT ATC ACC AAG-3'

Table S1. List of primers for saturation mutagenesis.

Supplemental References

S1. Dadonaite, B., Crawford, K.H.D., Radford, C.E., Farrell, A.G., Yu, T.C., Hannon, W.W., Zhou, P., Andrabi, R., Burton, D.R., Liu, L., et al. (2023). A pseudovirus system enables deep mutational scanning of the full SARS-CoV-2 spike. Cell *186*, 1263-1278.e20. 10.1016/j.cell.2023.02.001.

S2. Bloom, J.D., and Neher, R.A. (2023). Fitness effects of mutations to SARS-CoV-2 proteins. Virus Evol *9*, vead055. 10.1093/ve/vead055.

S3. Doud, M.B., and Bloom, J.D. (2016). Accurate measurement of the effects of all amino-acid mutations on influenza hemagglutinin. Viruses *8*, E155. 10.3390/v8060155.

S4. Walls, A.C., Park, Y.J., Tortorici, M.A., Wall, A., McGuire, A.T., and Veesler, D. (2020). Structure, function, and antigenicity of the SARS-CoV-2 spike glycoprotein. Cell *181*, 281-292.e6. 10.1016/j.cell.2020.02.058.
