# Supplementary material for: Functional and antigenic characterization of SARS-CoV-2 spike fusion peptide by deep mutational scanning
Source: Nat Commun. 2024 May 14;15:4056. doi: 10.1038/s41467-024-48104-8 (PMC11094058; doi:10.1038/s41467-024-48104-8)
Supplement: Supplementary file 3 — Reporting Summary [file 41467_2024_48104_MOESM3_ESM.pdf]

nature portfolio

Corresponding author(s): Lok-Yin Roy Wong
Last updated by author(s): Apr 15, 2024

Reporting Summary

Nature Portfolio wishes to improve the reproducibility of the work that we publish. This form provides structure for consistency and transparency in reporting. For further information on Nature Portfolio policies, see our Editorial Policies and the Editorial Policy Checklist.

Statistics

For all statistical analyses, confirm that the following items are present in the figure legend, table legend, main text, or Methods section.

n/a	Confirmed	
[ ]	[x]	The exact sample size (n) for each experimental group/condition, given as a discrete number and unit of measurement
[x]	[ ]	A statement on whether measurements were taken from distinct samples or whether the same sample was measured repeatedly
[ ]	[x]	The statistical test(s) used AND whether they are one- or two-sided<br>*Only common tests should be described solely by name; describe more complex techniques in the Methods section.*
[x]	[ ]	A description of all covariates tested
[x]	[ ]	A description of any assumptions or corrections, such as tests of normality and adjustment for multiple comparisons
[ ]	[x]	A full description of the statistical parameters including central tendency (e.g. means) or other basic estimates (e.g. regression coefficient) AND variation (e.g. standard deviation) or associated estimates of uncertainty (e.g. confidence intervals)
[ ]	[x]	For null hypothesis testing, the test statistic (e.g. F, t, r) with confidence intervals, effect sizes, degrees of freedom and P value noted<br>*Give P values as exact values whenever suitable.*
[x]	[ ]	For Bayesian analysis, information on the choice of priors and Markov chain Monte Carlo settings
[x]	[ ]	For hierarchical and complex designs, identification of the appropriate level for tests and full reporting of outcomes
[ ]	[x]	Estimates of effect sizes (e.g. Cohen's d, Pearson's r), indicating how they were calculated

Our web collection on statistics for biologists contains articles on many of the points above.

Software and code

Policy information about availability of computer code

Data collection	Pseudovirus luciferase activity was measured with BioTek Synergy H1 Hybrid Multi-Mode Microplate Reader. FoldX (v 5.0) was used for structural modeling. Illumina MiSeq PE250 was used for sequencing. Biolayer interferometry data was collected using an Octet RED96e System.
Data analysis	Custom python scripts were used for analyzing the deep mutational scanning data. Custom python scripts for all analyses have been deposited to: https://github.com/nicwulab/SARS2_FP_DMS<br>Data was analysed and graphed with GraphPad Prism 10 (v 10.2.0) and Microsoft Excel (v 16.82). R studio (v 2023.03.0-daily+82.pro2) was used for sequence data analysis and heatmap generation. Octet analysis software (v 9.0) was used for analyzing biolayer interferometry data.

For manuscripts utilizing custom algorithms or software that are central to the research but not yet described in published literature, software must be made available to editors and reviewers. We strongly encourage code deposition in a community repository (e.g. GitHub). See the Nature Portfolio guidelines for submitting code & software for further information.

Data

Policy information about availability of data

All manuscripts must include a data availability statement. This statement should provide the following information, where applicable:

- Accession codes, unique identifiers, or web links for publicly available datasets
- A description of any restrictions on data availability
- For clinical datasets or third party data, please ensure that the statement adheres to our policy

Raw sequencing data have been submitted to the NIH Short Read Archive under accession number: BioProject PRJNA910585. NMR structure of the bFP and FPPR were retrieved from PDB (7MY8). Raw data are provided in the "Source Data" file with this paper.

Research involving human participants, their data, or biological material

Policy information about studies with human participants or human data. See also policy information about sex, gender (identity/presentation), and sexual orientation and race, ethnicity and racism.

Reporting on sex and gender	N/A
Reporting on race, ethnicity, or other socially relevant groupings	N/A
Population characteristics	N/A
Recruitment	N/A
Ethics oversight	N/A

Note that full information on the approval of the study protocol must also be provided in the manuscript.

Field-specific reporting

Please select the one below that is the best fit for your research. If you are not sure, read the appropriate sections before making your selection.

☒ Life sciences ☐ Behavioural & social sciences ☐ Ecological, evolutionary & environmental sciences

For a reference copy of the document with all sections, see nature.com/documents/nr-reporting-summary-flat.pdf

Life sciences study design

All studies must disclose on these points even when the disclosure is negative.

Sample size	No sample size calculation was performed in advance. Numbers of mice analyzed were based on previous studies and on the numbers required to obtain statistical significance (PMID:35314834). Sample size for other experiments were based on the numbers required to obtain statistical significance.
Data exclusions	No data were excluded.
Replication	All in vitro findings were confirmed in at lest two independent experiments. All results can be reproduced. All in vivo experiments were repeated twice and data were pooled from independent experiments.
Randomization	Mice and cells were randomly assigned into each groups for all the experiments.
Blinding	Since all of the mouse studies were performed in the BSL3 laboratory, blinding was not possible because of biosafety considerations. For other experiments, blinding were not possible as the study was performed by multiple investigators at various locations.

Reporting for specific materials, systems and methods

We require information from authors about some types of materials, experimental systems and methods used in many studies. Here, indicate whether each material, system or method listed is relevant to your study. If you are not sure if a list item applies to your research, read the appropriate section before selecting a response.

Materials & experimental systems

n/a	Involved in the study
☐	☒ Antibodies
☐	☒ Eukaryotic cell lines
☒	☐ Palaeontology and archaeology
☐	☒ Animals and other organisms
☒	☐ Clinical data
☒	☐ Dual use research of concern
☒	☐ Plants

Methods

n/a	Involved in the study
☒	☐ ChIP-seq
☒	☐ Flow cytometry
☒	☐ MRI-based neuroimaging

Antibodies

Antibodies used	SARS-CoV-2 anti-N antibody (SinoBiological, Cat. #: Cat: 40143-R001);<br>CoV 44-79 and CoV 44-62 were expressed and purified inh-house as described in the Methods.<br>Rabbit polyclonal anti-SARS-CoV-2-S1 (SinoBiological, catalog #: 40591-T62);<br>Mouse anti-C9 (EMD Millipore, catalog #: MAB5356);<br>Mouse monoclonal anti-VSV-M (KeraFast, catalog #: EB0011);<br>HRP anti-mouse antibody (Thermo Fisher, catalog #: 31430);<br>HRP anti-rabbit (Thermo Fisher, catalog #: 31460)
Validation	Antibodies purchased from commercial vendor have been validated by the manufacturer for the specific application in this study. Experiments were performed under the conditions specified by the manufacturer. Antibodies produced in-house have been validated by neutralization assay with authentic SARS-CoV-2 and pseudoviruses.

Eukaryotic cell lines

Policy information about cell lines and Sex and Gender in Research

Cell line source(s)	HEK293T (ATCC, CRL-3216), Calu-3 (BEI Resources, NR-55340), Vero (ATCC, CRL-1586), Vero-TMPRSS2 (gift from Dr. Michael Diamond, Washington University in St. Louis) and Vero-TMPRSS2/ACE2 (BEI Resources, NR-54970)
Authentication	None of the cells were formally authenticated although Calu-3, Vero E6, Vero-TMPRSS2 and Vero-TMPRSS2/ACE2 cells remained sensitive to infection with SARS-CoV-2. 239T cells used supported antibody expression and pseudovirus entry.
Mycoplasma contamination	Negative for Mycoplasma
Commonly misidentified lines<br>(See ICLAC register)	No commonly misidentified cell lines were used.

Animals and other research organisms

Policy information about studies involving animals; ARRIVE guidelines recommended for reporting animal research, and Sex and Gender in Research

Laboratory animals	4 to 6 months old C57BL/6 mice. Mice were housed under standard conditions of dark/light cycle, ambient temperature and humidity.
Wild animals	No wild animals were used in this study
Reporting on sex	Both male and female were used in the study.
Field-collected samples	No field-collected samples were used in this study
Ethics oversight	All studies approved by the Institutional Animal Care and Use Committee at the University of Iowa (protocol #2071795-013)

Note that full information on the approval of the study protocol must also be provided in the manuscript.

Plants

Seed stocks	N/A
Novel plant genotypes	N/A
Authentication	N/A
